# Fatigue Shifts and Scatters Heart Rate Variability in Elite Endurance Athletes

**DOI:** 10.1371/journal.pone.0071588

**Published:** 2013-08-12

**Authors:** Laurent Schmitt, Jacques Regnard, Maxime Desmarets, Fréderic Mauny, Laurent Mourot, Jean-Pierre Fouillot, Nicolas Coulmy, Grégoire Millet

**Affiliations:** 1 National School of Mountain Sports/National Ski-Nordic Centre, Premanon, Les Rousses, France; 2 University of Franche-Comte, Research unit EA3920, “Prognostic markers and control factors in cardiovascular pathologies” and Physiology Department, University Hospital of Besançon, Besançon, France; 3 Clinical Research Methods Center, University Hospital of Besançon, Besançon, France; 4 University of Franche-Comte, Research unit UMR 6249 “Chrono-environment”, University Hospital of Besançon, Besançon, France; 5 University of Franche-Comte, Research unit EA4660 “Culture Sport Health Society and Exercise Performance Health Innovation platform”, UPFR des Sports 31 chemin de l’Epitaphe, Besançon, France; 6 University of Paris 13, Research unit EA2363, ARPE, 74 rue Marcel Cachin, Bobigny, France; 7 French Ski Federation, 50 rue des Marquisats, BP 2451, Annecy, France; 8 Institute of Sport Sciences, Department of Physiology, Faculty of Biology and Medicine, University of Lausanne, Lausanne, Switzerland; University Heart Center Freiburg, Germany

## Abstract

**Purpose:**

This longitudinal study aimed at comparing heart rate variability (HRV) in elite athletes identified either in ‘fatigue’ or in ‘no-fatigue’ state in ‘real life’ conditions.

**Methods:**

57 elite Nordic-skiers were surveyed over 4 years. R-R intervals were recorded supine (SU) and standing (ST). A fatigue state was quoted with a validated questionnaire. A multilevel linear regression model was used to analyze relationships between heart rate (HR) and HRV descriptors [total spectral power (TP), power in low (LF) and high frequency (HF) ranges expressed in ms^2^ and normalized units (nu)] and the status without and with fatigue. The variables not distributed normally were transformed by taking their common logarithm (log_10_).

**Results:**

172 trials were identified as in a ‘fatigue’ and 891 as in ‘no-fatigue’ state. All supine HR and HRV parameters (Beta±SE) were significantly different (P<0.0001) between ‘fatigue’ and ‘no-fatigue’: HR_SU_ (+6.27±0.61 bpm), logTP_SU_ (−0.36±0.04), logLF_SU_ (−0.27±0.04), logHF_SU_ (−0.46±0.05), logLF/HF_SU_ (+0.19±0.03), HF_SU_(nu) (−9.55±1.33). Differences were also significant (P<0.0001) in standing: HR_ST_ (+8.83±0.89), logTP_ST_ (−0.28±0.03), logLF_ST_ (−0.29±0.03), logHF_ST_ (−0.32±0.04). Also, intra-individual variance of HRV parameters was larger (P<0.05) in the ‘fatigue’ state (logTP_SU_: 0.26 vs. 0.07, logLF_SU_: 0.28 vs. 0.11, logHF_SU_: 0.32 vs. 0.08, logTP_ST_: 0.13 vs. 0.07, logLF_ST_: 0.16 vs. 0.07, logHF_ST_: 0.25 vs. 0.14).

**Conclusion:**

HRV was significantly lower in 'fatigue' vs. 'no-fatigue' but accompanied with larger intra-individual variance of HRV parameters in 'fatigue'. The broader intra-individual variance of HRV parameters might encompass different changes from no-fatigue state, possibly reflecting different fatigue-induced alterations of HRV pattern.

## Introduction

In elite sport, athletes training loads and recovery periods are managed to transitory disturb homeostasis and to subsequently reap a higher performance level [1]. This management has to avoid fatigue accumulation, which could abrade performance. As described by Meeusen et al. [2], the development of fatigue follows a continuum process ranging from voluntary and controlled fatigue necessary for performance progression and requesting few hours or few days of recovery, named functional over-reaching (FOR) [3], [4], until involuntary and uncontrolled fatigue requesting weeks or even months of recovery, named non functional OR (NFOR) [2] or overtraining (OT) [5] when it has become a “prolonged maladaptation”. In case of NFOR or OT, the increased recovery time results in a lack of training, a decrease of the physical capacities and finally an impaired performance [2]. Such an extreme fatigue can even cause the end of an athlete’s career.

The difficulty to distinguish FOR and specially NFOR from OT is well recognized [2], [6]. This is partially due to the fact that the multiple clinical signs of fatigue stem not only from training overload with inadequate rest but also from various inputs as psychological stress, nutritional imbalance, mild inflammatory disorders, etc… Several tools have been proposed to assess the fatigue level in daily routine. Among them, analysis of heart rate variability (HRV) provides an indirect evaluation of the heart control by the autonomic nervous system (ANS), and was highlighted as a promising tool [2]. Several studies attempted to unveil links between the training loads, the state of fatigue and the changes in ANS activity as reflected by HRV [7]–[14]. Unfortunately, the results of these studies were equivocal. In a case study of one overtrained cross-country skier, Hedelin et al. [11] reported reduced competition performance and lowered profile of mood states, along a decrease in absolute low frequency power (LF) while absolute HF power remained high. Conversely, Uusitalo et al. [12] showed that OT was associated with HRV descriptors in which HF power was decreased in nine female endurance athletes undergoing heavy training over a 6–9 week period. And Hedelin et al. [14] reported unchanged HRV values in nine overreached canoeists after increasing training load by 50% over a 6-day training camp, despite concomitant decreases in maximal blood lactate concentration, running time to fatigue, maximal and submaximal heart rate, as well as maximal oxygen uptake (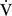
O_2max_).

These discrepant results might be related to several conditions. The former studies were conducted on quite short follow-up periods (few weeks or months in most cases) and involved a limited number of athletes (1 to ∼10–15). The protocols used were sometimes designed to artificially overload the athletes, without substantial evidence that a fatigue state had been induced. Another point of interest for the monitoring of athletes might be the assessment of HRV dispersion as shown recently in Plews et al. [15]. Therefore, if the different fatigue patterns quoted above were to be recognized according to different shifts in HRV parameters, then HRV parameters dispersion could reach broader ranges in ‘fatigue’ than in ‘no-fatigue’ states.

The present longitudinal study was conducted with records taken in ‘real life’ conditions. It aimed firstly at comparing HRV differences in elite Nordic skiers who were recognized either in a ‘fatigue’ or in a ‘no-fatigue’ state, independently of HRV recording, and secondly at measuring the fluctuation span of the HRV parameters in these two states. The hypotheses tested were firstly that HRV parameters of the athletes would be significantly different in the ‘fatigue’ and in the ‘no-fatigue’ states and, secondly that the dispersion of individual HRV parameters would be larger in the ‘fatigue’ state than in ‘no-fatigue’ condition.

## Methods

### Ethic Statement

The study submitted here was part of a larger project designed as “Hypoxic training in high level endurance athletes: evaluation of effects on the performance, research of individual responses, evaluation of potential risks for the health”. The whole project was approved by the Necker Hospital Ethic Committee (Paris, France). All the subjects provided written, voluntary, informed consent.

### Experimental Design

French elite Nordic skiers were involved in a 4-year follow-up. No ‘artificial’ change was applied to their training program. The fatigue state of the athletes was assessed by using the questionnaire designed and validated by the consensus group on overtraining of the French Society of Sports Medicine (QSFMS) (e.g. **Table S1**) [16]–[18].

The experiments were performed at the French Nordic-ski centre of Prémanon, France, between 2004 and 2008, by the same investigator, head physiologist of the Nordic ski teams, in French national teams of three disciplines: biathlon, Nordic-combined and cross-country skiing. HRV testing was part of the follow-up of the athletes during their yearly preparation for World cups and World championships and also during their Olympic preparation in 2006. During the 4-year study, the recordings of HRV were not prospectively scheduled, but the athletes were requested to regularly perform the HRV test, during every period of the training plan that can be described as: the general training period takes place in May and June and embeds base aerobic and strength training sessions; the specific training period follows with base aerobic, maximal aerobic and strength training sessions in July, August and September; the precompetitive period occurs in October and November and holds base aerobic, maximal aerobic and strength training sessions and altitude training camps. The competition period encompasses December to March, and April is the recovery period.

### Subjects

The subjects were 57 (27 men, 30 women) members of the French national teams of the Nordic ski disciplines and included 8 medallists of the Olympics Games, the World championships or the World cup. Inclusion criteria were as follows: elite athlete member of the French national teams from 2004–2005 to 2007–2008 training seasons with at least one declared fatigue state. So, only athletes with both ‘fatigue’ and ‘no-fatigue’ states were included retrospectively.

Exclusion criteria were to be injured or sick for longer than 5 consecutive days or to stop the international sporting career.

All these subjects had a maximum oxygen consumption (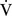
O_2max_) measurement at the entrance of the period of the study, all measured during an incremental treadmill test at the altitude of 1200 m as described earlier [19].

### HRV Tests

The tests were always performed in the same conditions. The subjects arrived at the national French training centre at 8h30 in the morning before the first training session of the day. All the athletes of one team completed together the HRV tests in the medical laboratory under supervision of the same investigator. No heavy training session took place during the two days preceding the HRV tests. During the competition period, the tests were performed on Wednesday morning, i.e. after two full days of easy aerobic training following the last competition run which usually take place Saturday and/or Sunday.

After a 15-min rest, the test began with 8 min supine (SU) followed by 7 min standing (ST). HRV analyses were performed on RR intervals recorded between the 3^rd^ and 8^th^ min supine, and between the 9^th^ and 14^th^ min standing. Measurement of the interval duration between two R waves of the cardiac electrical activity was performed with a HR monitor (T6, Suunto®, Vantaa, Finland). HRV assessment from these RR intervals has been validated against ECG measurements [20]. Each data file was visually inspected for artifacts, which were manually corrected. Then the spectral power was calculated with Fast Fourier Transform (FFT) according to the specific software (Nevrokard® HRV, Medistar, Ljubljana, Slovenia). Recording periods of 256 s were analysed, each yielding 512 data points after re-sampling at the 2 Hz frequency. The Hanning windowing function was applied and the Goertz algorithm was used for calculation. The power spectral density was measured by frequency bands in ms^2^.Hz^−1^ and the spectral power was expressed in ms^2^ [21] : High frequency (HF) power band (0.15 to 0.40 Hz) reflects the parasympathetic influence and is related to the respiratory sinus arrhythmia [22]; Low frequency (LF) power band (0.04 to 0.15 Hz) is also driven by parasympathetic tone, and is presently considered as carrying vagal resonances to either changes in vasomotor tone (often sympathetic) or in central modulation of sympathetic tone [23]; the spectral power in this frequency band has been found related to arterial blood pressure [22], [24] and to baroreflex activity [25].

During the tests, the investigator surveyed visually the breathing frequency and timed it with a frequency-meter, because a very low rate, under 9 cycles per min, could shift frequencies lower than 0.15 Hz and decrease the HF band or increase the LF band [26].

Both in supine (SU) and in standing (ST) positions, LF and HF were calculated in absolute spectral power units (ms^2^) and in normalized units (nu) with LF(nu) = LF/(LF+HF)×100 and HF(nu) = HF/(HF+LF)×100. The total spectral power (TP) was calculated by adding LF and HF. The LF/HF ratio was also calculated as an indicator of sympathetic over parasympathetic balance [21].

### Definition of the State of Fatigue

The states of fatigue were identified according to the scoring at the QSFMS (**Table S1**), which was fulfilled after the RR interval recording procedure. This questionnaire is used routinely in French national teams in various sports (rugby, swimming, Nordic-ski, triathlon…). It has been built up by the consensus group on overtraining of the French Society of Sports Medicine, a large panel of scientists and sport physicians. This standardized description of the psychological/behavioural state takes into account different contributions to fatigue linked to physical exercising. A score calculated from the answers to the QSFMS quantifies the clinical symptoms of overtraining. The state of fatigue was registered when the score exceeded 20 negative items out of 54 [16], [17]. This score was shown to correlate with indications of muscular damage (creatine kinase, myosin) and some hematological variables (blood viscosity, hematocrit, plasma viscosity, ferritin) [16].

### Statistical Analysis

The ‘Fatigue’ variable was dichotomized according to the threshold of 20 items with a negative answer. In each recording position (SU and ST) of RR intervals the following variables describing heart rate variability (HRV) were quantified. HR_SU_, HR_ST_, LF_SU_(nu), HF_SU_(nu), LF_ST_(nu), HF_ST_(nu) were assumed to be distributed normally. The remaining variables were transformed by taking their common logarithm (log_10_).

The relationships between the HRV parameters and the state of ‘fatigue’/‘no-fatigue’ variable were analyzed using a multilevel linear regression model with a complex variance structure. In this model, level 1 was the rank of the measure and level 2 the subject. The rank (for one given subject, each successive rank labelled a recording date) of the measure was used as an orthogonal polynomial in order to account for repeated measurements. Each HRV variable was first analyzed in a univariate model. Gender and age of the subjects were introduced in the statistical model. A random parameter was finally introduced at level 1 in order to control for heteroscedasticity of the HRV parameters. This random parameter allowed assessing differences in HRV power dispersion between ‘fatigue’ and ‘no-fatigue’ states in each subject. Normal distribution of the residuals was checked on final models. Significance threshold was set to 0.05. Analyses were performed using MLwiN V2.20 [27]. These models allowed analyzing unbalanced data, as the number of recordings for each subject.

## Results

The characteristics of the subjects as they entered the study are shown in **Table 1**. The values of 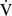
O_2max_ were consistent with the elite level of the subjects.

**Table 1:** Anthropometric characteristics and 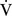
O_2max_ of the subjects at the time of their inclusion in the study.

	Gender (n)	Age (years)	Weight (kg)	Height (cm)	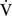 O_2max_ (mL.min^−1^.kg^−1^)
Biathlon	9 m	22.6±4.0	71.8±5.2	180.2±4.8	78.0±5.0
Biathlon	18 w	23.7±4.1	58.5±6.1	167.4±4.7	58.1±4.4
NC	13 m	22.7±4.3	63.4±5.2	176.2±5.5	67.7±4.5
XCS	5 m	20.4±0.9	73.2±2.7	183.0±3.2	79.8±3.2
XCS	12 w	23.2±3.7	58.0±3.8	166.3±2.6	56.6±3.8
**All**	**57**	**22.9±3.9**	**62.9±7.7**	**172.6±7.6**	**65.1±10.0**

NC = Nordic-combined. XCS = Cross country skiing. m = men, w = women.

### QSFMS

**Figure 1:**
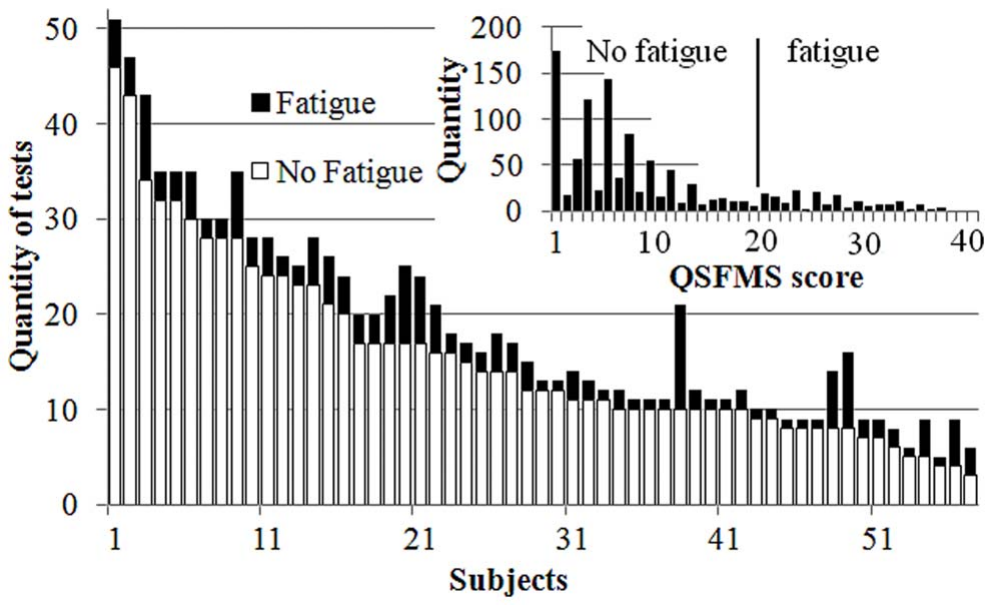
Number of HRV tests recorded in ‘No-fatigue’ and ‘Fatigue’ state in each subject, and overall distribution of the QSFMS scores. In each subject’s column the HRV recordings obtained when the QSFMS had a score under the 20-item threshold are in white, and those recorded during a scored state of fatigue are in black.

The distribution of the QSFMS scores is displayed in **Figure 1**. The average score of the 1063 tests performed was 8.84 (Standard deviation, SD = 8.94). 172 tests had a score higher than 20 indicating a ‘fatigue’ state, which amounted to 16.2% of all the tests performed. **Figure 1** also displays the distribution of the number of tests performed, and for every athlete indicates the number of ‘fatigue’ and ‘no-fatigue’ states diagnosed with the QSFMS.

### HRV Analysis

During the tests, the breathing frequencies (BF) were recorded and no breath holding period was observed.

Supine and standing BF data in ‘fatigue’ and ‘no-fatigue’ states are displayed in **Table 2** **.**

**Table 2:** QSFMS, Breath Frequency (BF), Heart Rate (HR) and parameters of heart rate variability (HRV): distribution of values across the ‘no-fatigue’ and ‘fatigue’ states.

			mean	min	10e	25e	median	75e	90e	max	P value*
**Supine**	**QSFMS**	No F	5.5	0.0	0.0	2.0	5.0	8.0	13.0	19.0	
	score	F	26.2	20.0	20.0	22.5	25.0	29.5	33.0	37.0	<0.0001
	**BF_SU_**	No F	12.5	9.0	10.0	11.0	12.0	14.0	15.0	18.0	
	(bpm)	F	13.9	9.0	11.0	12.0	13.0	16.0	17.0	21.0	<0.0001
	**HR_SU_**	No F	55.3	33.9	44.7	49.0	55.0	61.0	66.5	92.0	
	(bpm)	F	63.3	41.0	48.6	55.4	62.8	68.1	78.3	99.0	<0.0001
	**LF_SU_**	No F	2398	66	458	805	1553	2874	5174	33496	
	(ms^2^)	F	1636	12	86	296	846	1864	3602	27133	<0.0001†
	**HF_SU_**	No F	3748	71	790	1411	2687	4563	8089	41526	
	(ms^2^)	F	2286	14	66	253	837	2489	5943	29989	<0.0001†
	**TP_SU_**	No F	7779	356	2026	3450	5782	10097	16352	50360	
	(ms^2^)	F	4942	67	252	847	2548	5532	12567	57500	<0.0001†
	**LF/HF_SU_**	No F	0.84	0.06	0.20	0.34	0.63	1.07	1.66	5.85	
		F	1.30	0.08	0.25	0.55	1.02	1.70	2.70	6.92	<0.0001†
	**LF_SU_**	No F	39.38	5.35	16.41	25.45	38.75	51.60	62.36	85.40	
	(nu)	F	48.98	7.58	19.96	35.69	50.58	62.95	72.95	87.37	<0.0001
	**HF_SU_**	No F	60.61	14.58	37.64	48.38	61.24	74.55	83.59	94.65	
	(nu)	F	51.00	12.63	26.97	37.05	49.42	64.31	80.04	92.42	<0.0001
**Standing**	**BF_ST_**	No F	15.9	10.0	13.0	14.0	15.0	18.0	20.0	23.0	
	(bpm)	F	17.6	10.0	14.0	15.0	17.0	20.0	22.0	25.0	<0.0001
	**HR_ST_**	No F	77.27	43.38	60.0	68.73	77.0	85.66	93.84	109.0	
	(bpm)	F	87.93	55.0	68.10	79.78	87.98	97.25	105.08	138.0	<0.0001
	**LF_ST_**	No F	3260	147	627	1108	2286	4133	6910	38881	
	(ms^2^)	F	1619	26	187	426	1050	2338	4183	8960	<0.0001†
	**HF_ST_**	No F	823	9	86	191	420	925	1908	18167	
	(ms^2^)	F	340	1	35	70	165	446	754	2892	<0.0001†
	**TP_ST_**	No F	6386	400	1413	2563	4753	8196	13604	53112	
	(ms^2^)	F	3223	77	389	1052	2108	4511	7266	21311	<0.0001†
	**LF/HF_ST_**	No F	7.23	0.22	1.68	3.03	5.56	9.68	14.70	52.18	
		F	7.79	0.21	2.09	3.42	5.73	9.75	14.34	44.00	0.32
	**LF_ST_**	No F	80.27	17.66	61.00	74.43	84.50	90.61	93.59	98.12	
	(nu)	F	81.14	4.02	66.65	75.65	84.84	90.39	93.48	97.78	0.43
	**HF_ST_**	No F	19.69	1.88	6.36	9.38	15.49	25.43	39.00	82.34	
	(nu)	F	18.84	2.22	6.52	9.61	15.12	24.35	33.35	95.98	0.43

No F = No Fatigue state n = 891. F = Fatigue state n = 172.

Min = minimum; 10^e^, 25^e^, 75^e^, 90^e^ = respectively tenth, twenty-fifth, seventy-fifth and ninetieth percentiles; QSFMS score = number of negative items (i.e.); SU = supine; ST = standing; BF = breath frequency (breaths per minute); HR = heart rate (beats per minute); LF = spectral power in the low frequency band; HF = spectral power in the high frequency band, TP = total spectral power; nu = normalized units.

* Multilevel analysis.

† The modeling was conducted on Log transformation.

The table describes pooled data of every athlete of the study. Thus, the differences between ‘fatigue’ and no-fatigue states are not directly comparable to statistical differences issued from the multilevel analysis in **Table 3**, which assessed intra-individual changes.

**Figure 2:**
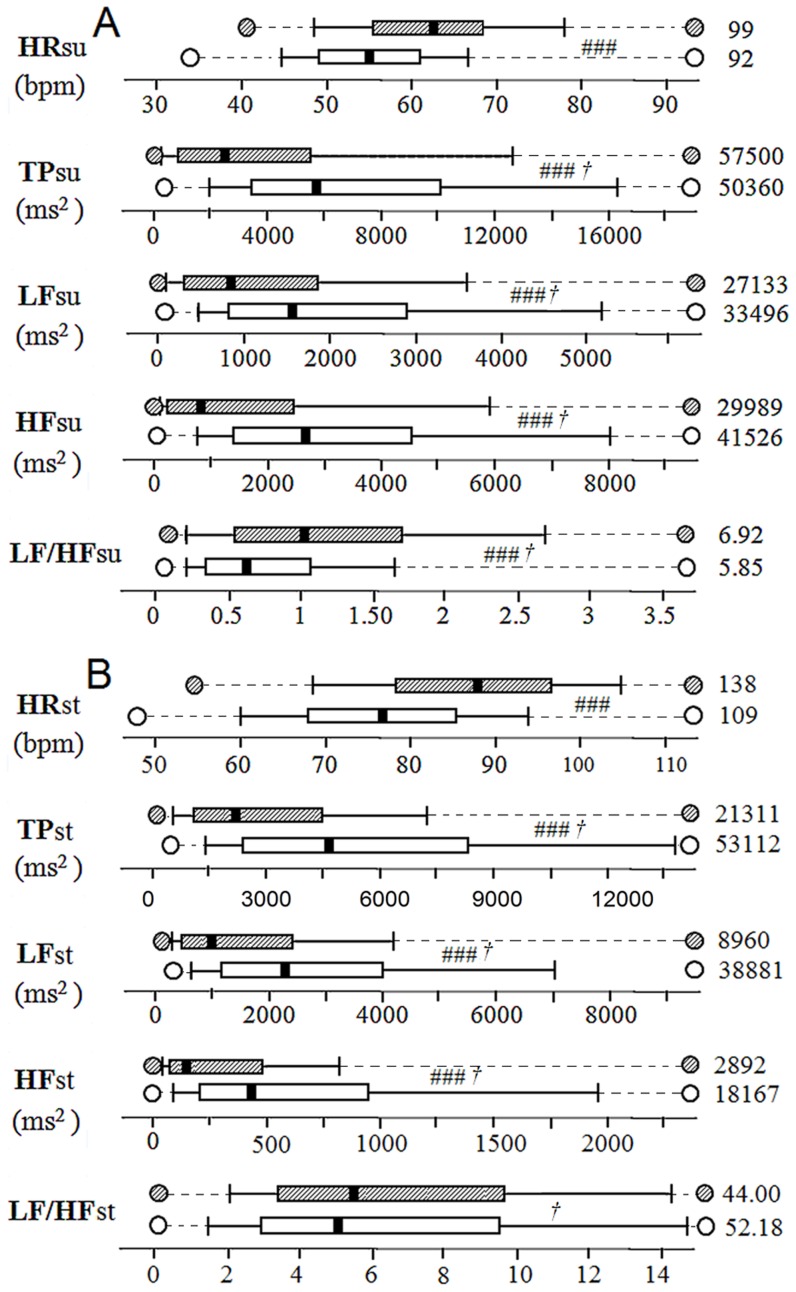
Distribution of values in heart rate and HRV parameters according to ‘fatigue’ and ‘no-fatigue’ states in supine (A) and standing (B) positions. The box and whiskers display values of all recordings for all the subjects. Box is defined by the first and third quartiles. The thick line stands for the median value. The tenth and ninetieth of values are marked by the “wisker” bars, and the circles stand for the lowest and highest values. The highest values are out of scale and the number is written at the right end of the bar close to the circle that should stand out of scale. The dotted dark line symbolizes a discontinued scale. The ‘fatigue’ states are in grey and the ‘no-fatigue’ states are in white. ‘no-Fatigue’ state n = 891, ‘fatigue’ state n = 172. SU = supine position; ST = standing position; HR = heart rate (beat per minute); TP = total spectral power (ms^2^); LF = spectral power in the low frequency band (ms^2^); HF = spectral power in the high frequency band (ms^2^). Right figure is the highest value of the file. † Analysis was conducted on Log transformed data. ### for p<0.001 in differences between ‘fatigue’ and no-fatigue state using multilevel models. The scaling was chosen for a clear display of figures span of each parameter. Therefore scales are different between parameters and between supine and standing positions. It has to be noticed that the distribution of values displayed in this figure are computed from all the recordings of every subject cannot be directly compared with the intra-individual variances assessed by the multi-level analysis (Table 3).

**Table 2** and **Figure 2** describe the distribution of values of HRV parameters recorded in all the subjects both the ‘no-fatigue’ and ‘fatigue’ states. **Table 3** displays the results of the multilevel linear regression models with the intra-subject variances.

**Table 3:** HRV parameters across ‘fatigue’ and ‘no-fatigue’ state: multilevel linear modelling.

		Var X = fatigue			Intra-subject variance		
	Var Y	Beta	SE	p value	No-fatigue	Fatigue	p value
**Supine**	**HR (bpm)**	6.27	0.61	<0.0001	31.86	53.09	<0.0006
	**logLF**	−0.27	0.043	<0.0001	0.11	0.28	<0.0001
	**logHF**	−0.46	0.05	<0.0001	0.08	0.32	<0.0001
	**logTP**	−0.36	0.04	<0.0001	0.07	0.26	<0.0001
	**log LF/HF**	0.19	0.03	<0.0001	0.08	0.09	0.45
	**LF (nu)**	9.55	1.33	<0.0001	201.82	243.89	0.15
	**HF (nu)**	−9.55	1.33	<0.0001	201.75	243.86	0.15
**Standing**	**HR (bpm)**	8.82	0.89	<0.0001	71.44	115.37	<0.001
	**log LF**	−0.29	0.03	<0.0001	0.07	0.16	<0.0001
	**logHF**	−0.32	0.04	<0.0001	0.14	0.25	<0.0001
	**logTP**	−0.28	0.03	<0.0001	0.07	0.13	<0.0001
	**log LF/HF**	0.03	0.03	0.32	0.09	0.10	0.88
	**LF (nu)**	0.79	0.99	0.43	127.41	131.51	0.80
	**HF (nu)**	−0.79	0.99	0.43	126.44	131.58	0.75

The relationships between HR or HRV parameters and the fatigue variable (X variable) were analysed separately: one model for each HR or HRV parameters (Y variables) and each line of the table presents the results of one model. Beta column displays the model parameter and can be seen as the average distance between the values of the ‘fatigue’ and the ‘no-fatigue’ states, as e.g. in ‘fatigue’ recordings supine HR values were on average 6.27 bpm higher and logTP of HRV were on average 0.36 lower than when measured with ‘no-fatigue’ QSFMS score. The variance columns display the within–subject variance of values across the ’no-fatigue’ and ‘fatigue’ state variable (including significance probability).

In supine position, the HR values were on average 6.27 bpm higher in recordings performed in the ‘fatigue’ state than when there was ‘no-fatigue’ (P<0.0001). Conversely, the HF power (nu) was on average 9.55 lower in the ‘fatigue’ than the ‘no-fatigue’ (P<0.0001). Thus, in ‘fatigue’ state, logTP_SU_, logLF_SU_, logHF_SU_, and HF_SU_(nu) were significantly lower than in ‘no-fatigue’ state (all P<0.0001). Conversely, logLF/HF_SU_ was larger in ‘fatigue’ state (P<0.0001). In standing position, HR_ST_ was higher and logTP_ST_, logLF_ST_, logHF_ST_ were lower with fatigue than without (P<0.0001).

The intra-subject HR variances were larger in ‘fatigue’ than ‘no-fatigue’ state in the supine position (respectively: 53.09 vs 31.86; P<0.0006) and in the standing position (respectively 115.37 vs 71.44; P<0.001). Intra-subject variance was also larger in fatigue state for logTP_SU_, logLF_SU_, log HF_SU_, logTP_ST_, logLF_ST_, logHF_ST_ (P<0.0001). These statistically significant results are thus not reflected in the group description displayed in **Figure 2**.

The model did not reveal any difference linked to gender or age of the subjects.

**Figure 3:**
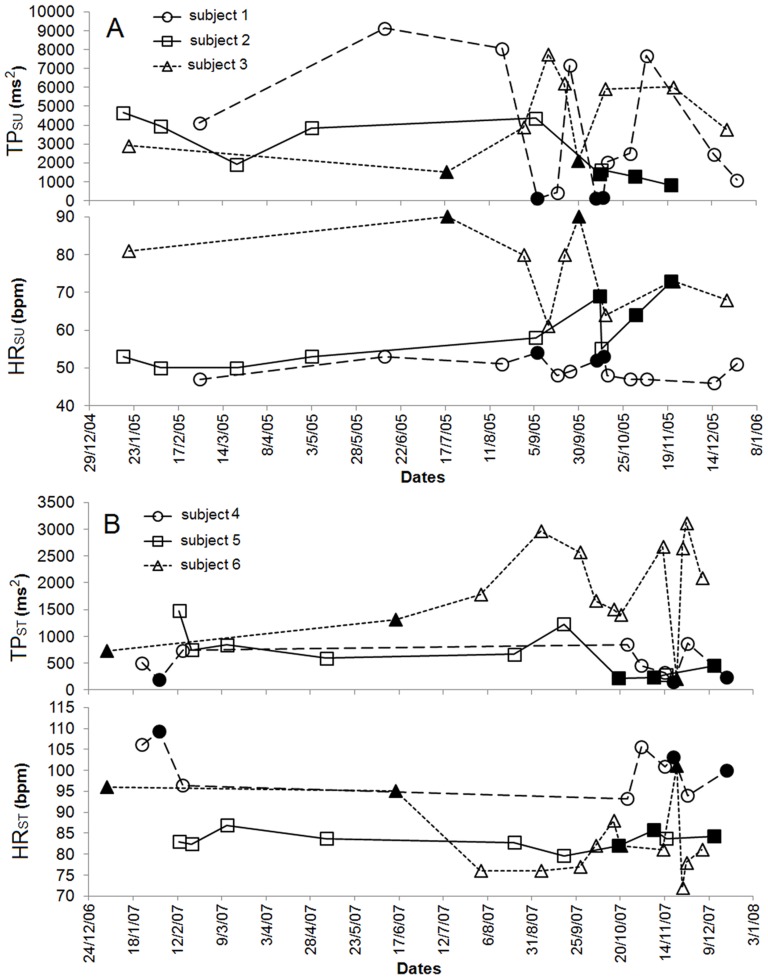
Cases examples of HRV analysis monitoring from individual measurements in ‘fatigue’ and ‘no-fatigue’ states in supine (A) and standing (B) positions. For three subjects in supine (A) and three other in standing (B) positions, monitoring of total spectral power (TP) by adding low and high frequencies in absolute units (ms^2^) and heart rate (HR) (bpm) during the same surveyed year. ‘no-fatigue’ points are in white and “fatigue’ points in black.

**Figure 3** displays examples of HRV analysis monitoring from individual measurements in ‘fatigue’ and ‘no-fatigue’ states in supine and standing positions.

## Discussion

This study surveyed elite endurance athletes over a multi-year period without any ‘artificial’ (e.g. study-related) change of their training regimen. It led to two main results. Firstly, as hypothesized, most of the HRV parameters were different in ‘fatigue’ vs. ‘no-fatigue’ states. These differences were observed both in supine (HR, LF/HF and LFnu increased; LF, HF, TP, HFnu decreased in ‘fatigue’) and in standing (HR increased; LF, HF and TP decreased in ‘fatigue’) positions. Secondly, the intrasubject variance of HR, LF, HF and TP was significantly larger in ‘fatigue’ than in ‘no-fatigue’ state. This suggests that different fatigue-shifted HRV patterns coexist in the ‘fatigue’ state.

Most studies investigating the relationships between fatigue (e.g. during states labelled as OR (FOR or NFOR) or OT [2]) and HRV embedded methodological features that limit the impact of their findings. Indeed, most studies enrolled few athletes (1 to 10–15; e.g.; Hedelin et al. [11], [14]; Uusitalo et al. [12]). Considering the high inter-individual variation in the response to training and fatigue, the small subject number largely increases the risk of statistical errors. Also, in some studies the usual training regimen of the subjects was modified by artificially introducing an overload period [8], [12], which makes it difficult to ascertain that a true state of fatigue is achieved. Moreover, some of the previously published studies did not clearly describe the criteria used to separate the ‘fatigue’ and ‘no-fatigue’ states [11], [28]. Finally, the fact that physiological data recorded or investigated in sub-elite [28] or well-trained subjects cannot be translated to elite level is also of importance [29].

While HRV recording tests were not performed as many times in every athlete, we used an appropriate statistical approach. The statistical multilevel model was not affected by the non balanced number of recordings in subjects [30]. In addition the model coped with heteroscedasticity, i.e. the non homogenous variance of HRV parameters across the two states of the ‘fatigue’ independent variable. The conditions and procedure of data recording were also greatly standardized, i.e. same location (medical laboratory); same timing in the day and the week; same recording device; same investigator; routinely implemented HRV recordings. This likely contributed to reduce misleading causes of variance in results.

HRV values were obtained before the subject completed the QSFMS questionnaire (**Table S1**). The questionnaire data were analyzed afterward and blindly from the HR recording. Thus quoting a ‘fatigue’ state was done independently of HRV analysis. In this study, quoting a ‘fatigue’ state according to the threshold of 20 negative items out of 54 was the only criterion for statistical comparison of HRV data in a model that takes into account potential intra-subject pairing of data between ‘fatigue’ and ‘no-fatigue’ states.

In a comprehensive review on prevention, diagnosis and treatment of the overtraining syndrome, Meeusen et al. [2] highlighted the importance of the diagnosis and follow-up of the fatigue changes in elite sport. The authors reported the pros and cons of different methods currently used; e.g. analysis of the hormonal status (testosterone/cortisol ratio, ACTH/cortisol ratio, growth hormone, insulin-like-growth-factor-I), of the performance level, of the psychological and mood state (Profile of Mood State, POMS; Recovery-Stress Questionnaire, RestQ-Sport or the Daily Analysis of Life demands of Athletes, DALDA), of the immune function, and finally of the HRV. It is largely recognized that a single biological marker is only very seldom determinant in diagnosing a fatigue state. When this occurs, several other symptoms -and other biological signs- are most often gathered to highlight a severe fatigue state. Such a recognition happens often late in the course of the athlete’s follow-up. Obtaining biological values requires blood sampling and/or urine sampling, is invasive and costly, and hence cannot be repeated frequently, i.e., as frequently as requested in monitoring elite athletes in whom extended impairment of function should be avoided. HRV recording is just the opposite on several points, and therefore it could be easily used in daily routine [2]. The idea of fatigue encompasses more than overtraining. Fatigue likely comprises different kinds of impairments not only triggered by the imbalance of training load and recovery, but also by e.g. infectious episode, impaired sleep (which can be a symptom of fatigue) or emotional/affective insult… [2]. All these conditions can lead to some physiological imbalance likely to involve some shift in autonomic settings, and finally some cardiac HRV change.

Using the 20 items threshold of the QFSMS questionnaire (**Table S1**) to separate fatigue and no-fatigue records (**Figure 1**), the multilevel linear regression model used in this study led to significant differences between the ‘fatigue’ and ‘no-fatigue’ states in most HRV descriptors (**Table 3**). On average HR was higher and HRV was lower during fatigue than in no-fatigue state both supine and standing (**Table 2** and **Figure 2**). Less of autonomic inputs or resonances to physiological signals imply less accurate autonomic controls. Hence some loss of efficiency is likely in metabolic and physiological functions, which are instrumental to sharp sport performance. The lowering of HR autonomic control would thus appear as an easily discernible cue of more complex and widely spread impairments in fatigue states. All the frequency band descriptors were lowered in fatigue state. In supine position, the power decrease was lesser in LF than HF, leading to a higher LF/HF ratio and suggesting a less parasympathetic control on HR. The changes in LFnu and HFnu also displayed this pattern. The simultaneous decrease of total spectral power and the larger contribution of LF variability were thus in line with previous reports in fatigue after competition [31] overreaching [32], [33] or overtraining states [8], [10], [12]. In the standing position, the ‘fatigue’ state was associated on average with decreased TP, LF and HF powers and increased HR; but the values of LF/HF ratio and LF(nu) were not significantly different from the ‘no-fatigue’ state. The methodology used in this study does not allow searching for any particular state or cause of fatigue.

The increase in heart rate would be simply explained in a reciprocal activation pattern of autonomic arms [34]. Indeed the higher HR in fatigue state leads to the idea of a decreased vagal influence also possibly associated with an increased sympathetic activation. As all absolute HRV variables (TP, LF and HF spectral powers) are mainly under vagal modulation and are lowered, a lessened vagal modulation of heart activity is most likely when fatigued. The observation of higher LF/HF at supine rest supports the idea that a balanced autonomic control has shifted towards sympathetic predominance. A co-activation of vagal and sympathetic outflows might bring opposite changes in HR and in the relationship between the dynamics of HR and LF/HF ratio [34], [35]. Such a co-activation of autonomic arms is thus very unlikely in the present results. A further detailed recognition of different patterns of changes in spectral powers is difficult, and the assessment of potential linkages between such successive patterns requires models of data analysis that are beyond the scope of this study.

Significant differences in supine LF/HF and normalized LF and HF between No-Fatigue and Fatigue, but not in standing figures might be questioning. We believe this discrepancy is due to the following points. Firstly, the normalized values depend on the total power of HRV, which is largely lowered in fatigue states, and reduces in turn the scaling of normalized indices. Secondly, LF power holds much of parasympathetic influence. The reduction of HF power in fatigue states thus influences both terms of the ratio LF/HF and narrows its changes. Thirdly, since standing reduces total power and HF power in No-Fatigue state, and this physiological response occurs also during Fatigue states, the statistical assessment of significant further change in LF/HF due to fatigue becomes less likely (as reflected in median and mean figures). In standing position, the LF power likely embeds a larger vascular (e.g. pulse pressure) resonance than in supine position, which accounts for the higher LF/HF ratio in upright position. LF/HF value is more complexly determined than the quite simple ‘gold-standard’ criterium sometimes considered in HRV fatigue studies [23], [25]. We believe also that, although the comparison of standing and supine values is more complex in the fatigue than the ‘No-Fatigue’ state, taking it into account adds insight into HRV changes caused by ‘Fatigue’. Relative variables such as LF/HF ratio and “normalized” power might be useful, but each also presents a “constructed” view of data, easily confounding if not considered in the frame of the primary data.

Beside the overall differences in HRV parameters in ‘fatigue’ vs. ‘no-fatigue’ states, another finding of this study was the broader spanning of intra-subject HRV values in the fatigue state, as depicted by “variance” figures in the statistical model (**Table 3**). To the best of our knowledge, this has not been reported previously. The dispersion of values of HRV parameters between subjects (e.g. between-subject variance) is known to depend on age, gender, heritability, environmental factors such as noise, temperature, light [21], but the statistical analysis did not reveal any difference linked to gender or age of the subjects in our study. The dispersion of values of HRV parameters between athletes might also depend on the level of practice, the amount and type of physical activity. Such influences have been reported in normal or sedentary population [36]–[38]. Also, while monitoring the usual training course of such elite athletes, HRV tests are hardly obtained in exactly similar training conditions, particularly as regarding training load. In the present study, methodological precautions were taken to standardize the RR recordings used to HRV analysis. The subjects were homogenous regarding fitness and a several years training level. Also, as already stated, the statistical analysis relied on a model in which each subject was taken as his own reference, and to avoid potential confusion bias adjustment on a particular variable was performed when required. Therefore we believe that the shifts in quantitative repartition of HRV values are meaningful.

In sedentary subjects, Kleiger et al. [39] have reported the substantial variation in HRV values in fourteen normal subjects aged 20 to 55 years. Noritake et al. [40] found a larger diurnal variation in HRV parameters in nineteen healthy subjects than in twenty-nine diabetic patients. Similarly, Plews et al. [15] reported recently a case study of an elite triathlete in whom the day-to-day variation in the square root of the mean sum of the squares differences between R-R intervals (RMSSD), a temporal marker of the parasympathetic influence, was decreased in an OR state. This narrowed fluctuation of HRV when overreached seems conflicting with our results. However, we believe this discrepancy is mainly due to the fact that in the present study the broader span of values of HRV parameters in the ‘fatigue’ state encompasses different patterns of fatigue-induced changes in HRV spectra, which can display different or opposite shifts within the HRV spectrum. The study was not aimed at analyzing day-to-day variability but to highlight the complex content of HRV analysis. By the way, we support the point expressed in the paper by Plews et al. [15] that a decrease in day-to-day fluctuation of HRV parameters over a short period of time might help diagnosing the onset of overreaching. Also, a diversity of types of ‘fatigue’ could explain the higher intra-individual HRV variance when compared to ‘no-fatigue’ state, although each single ‘fatigue’ condition over few days can be stable or tend towards no response with the ‘law of initial values’ described in Plews et al. [15].

Our results may suggest that different and sometimes opposite autonomic regulatory influences could be explained by different degrees or states of fatigue, such as OR or OT [2]. The point of transition between these states is not precisely defined in the literature. This would explain why previous studies have reported equivocal findings, with increases [11], decreases [12] and no change [13], [14] in HRV power during OR or OT states. The greater variance of HRV figures in elite athletes in ‘fatigue’ state could suggest that different fatigue-shifted HRV patterns were simultaneously considered in this study, which is an entangled situation. Assessment of different degrees and sub-categories of fatigue and of the effectiveness of HRV in their recognition in elite athletes, require other kind of statistical models. Finally, the QSFMS questionnaire (**Table S1**) was validated to highlight a state of fatigue but it is possible that it only allows distinguishing the ‘fatigue’ state from ‘no-fatigue’ state. As far as we know, this questionnaire does not allow to distinguish sub-categories of fatigue.

### Limits and Strengths of the Study

As most ‘field’ studies, this retrospective longitudinal study has limits: **i)** The frequency of records was different between the athletes due to several factors: variable presence in the National centre; competition schedule (different between the Nordic disciplines and between men and women); examinations; illnesses… However, the statistical method was chosen to cope with this non balanced individual number of HRV recordings. **ii)** The quantification of objective performance is difficult in the Nordic ski disciplines since velocity is largely influenced by the environmental conditions (elevation, quality of snow, weather…). Thus a performance quotation valid enough to enhance the fatigue assessment performed with the QSFMS questionnaire would be difficult to implement. **iii)** Within the two days preceding HRV recording, the training content was strictly aerobic although it might individually fluctuate in volume, and thus somewhat influence the ensuing HRV values.

However, we believe that this study is unique for several reasons: i) The sample size is unequalled in a study involving elite athletes. ii) The very long period of investigation likely counterbalances some seasonal variations. iii) The reproducibility of HRV recordings and their technical quality were satisfying: same location (medical laboratory); same timing in the day and the week; same device; same investigator; routinely implemented HRV recordings. iv) The assessment of the states of ‘fatigue’ and ‘no-fatigue’ was based on a widely used tool and on an *a priori* threshold independent of HRV analysis. v) From a practical point of view, we consider the present study established a first step in relevance of frequency-domain HRV analysis for monitoring the fatigue in elite endurance athletes. It is at this stage difficult to speculate how various influences combine to enhance or decrease parasympathetic drive or resonance to sympathetic central or vasomotor control as carried in vagal efference to the heart.

### Conclusion

Values of HRV descriptors were significantly lower in ‘fatigue’ than ‘no-fatigue’ states in elite endurance athletes, both in supine and standing positions, over a multi-year period and without any ‘artificial’ change in their training regimen. The main trend by far was a ‘fatigue’-linked lowering of total HRV power as well as in the LF and HF frequencies bands. In addition, the intra-subject variance of values of HRV descriptors was larger in ‘fatigue’ than in ‘no-fatigue’ state. These differences strongly suggest that the ‘fatigue’ state encompassed differently-oriented shifts of HRV pattern, possibly reflecting differently-organized autonomic responses to “fatigue”. Further evaluation is required to assess how a thorough HRV analysis might help to distinguish between hypothetical different fatigue patterns.

## Supporting Information

Table S1 **Questionnaire of the French Society of Sport Medicine (QSFMS).**
(DOC)
