# Supplementary material for: Fatigue Shifts and Scatters Heart Rate Variability in Elite Endurance Athletes
Source: PLoS One. 2013 Aug 12;8(8):e71588. doi: 10.1371/journal.pone.0071588 (PMC3741143; doi:10.1371/journal.pone.0071588)
Supplement: Table S1 — Questionnaire of the French Society of Sport Medicine (QSFMS). (DOC) [file pone.0071588.s001.doc]

**Table S1: Questionnaire of the French Society of Sport Medicine (QSFMS)**

| 1 | My level of sport performance/my state of form has decreased: | Yes | No |
| --- | --- | --- | --- |
| 2 | I am not as attentive as before: | Yes | No |
| 3 | My close friends think that my behaviour has changed: | Yes | No |
| 4 | I have a sensation of oppression in my chest: | Yes | No |
| 5 | My heart seems to beat faster: | Yes | No |
| 6 | I have a lump in my throat: | Yes | No |
| 7 | I have less appetite than before: | Yes | No |
| 8 | I eat more: | Yes | No |
| 9 | I do not sleep as well as before: | Yes | No |
| 10 | I drowse and yawn in the daytime: | Yes | No |
| 11 | The lapse of time between training sessions seems to me too short: | Yes | No |
| 12 | My sexual appetence has decreased: | Yes | No |
| 13 | My performances are poor: | Yes | No |
| 14 | I frequently catch a cold: | Yes | No |
| 15 | I have put on weight: | Yes | No |
| 16 | I have memory problems: | Yes | No |
| 17 | I often feel tired: | Yes | No |
| 18 | I underestimate myself: | Yes | No |
| 19 | I often have cramps, muscular pain: | Yes | No |
| 20 | I suffer from headaches more frequently: | Yes | No |
| 21 | I do no feel fit: | Yes | No |
| 22 | I sometimes feel dizzy, on the point of fainting: | Yes | No |
| 23 | I do no confide in others so easily: | Yes | No |
| 24 | I am often seedy: | Yes | No |
| 25 | I have a sore throat more often: | Yes | No |
| 26 | I feel nervous, insecure, anxious: | Yes | No |
| 27 | I do no bear training so well: | Yes | No |
| 28 | At rest, my heart rate is faster than before: | Yes | No |
| 29 | During exercise, my heart rate is faster than before: | Yes | No |
| 30 | I often feel rotten: | Yes | No |
| 31 | I get tired more easily: | Yes | No |
| 32 | I often have digestive disorders: | Yes | No |
| 33 | I feel like staying in bed: | Yes | No |
| 34 | I am not so confident in myself: | Yes | No |
| 35 | I get injured more easily: | Yes | No |
| 36 | I have more difficulties in organizing my thoughts: | Yes | No |
| 37 | I have more difficulties in concentrating in my sports activity: | Yes | No |
| 38 | My sporting gestures are less precise, less skilful: | Yes | No |
| 39 | I have lost force and aggressiveness: | Yes | No |
| 40 | I feel as if I had no one to talk to: | Yes | No |
| 41 | I sleep longer: | Yes | No |
| 42 | I cough more often: | Yes | No |
| 43 | I do not enjoy practicing my sports as much: | Yes | No |
| 44 | I do not enjoy my leisure activities as much: | Yes | No |
| 45 | I get irritated more easily: | Yes | No |
| 46 | I am less efficient in my school or professional activity: | Yes | No |
| 47 | People around me think that I have become less pleasant: | Yes | No |
| 48 | Training seems harder and harder: | Yes | No |
| 49 | It is my fault if my results are worse: | Yes | No |
| 50 | My legs feel heavy: | Yes | No |
| 51 | I lose my personal things more easily (wallet, keys, etc.): | Yes | No |
| 52 | I am pessimistic, I have the blues: | Yes | No |
| 53 | I have lost weight: | Yes | No |
| 54 | My motivation, will and tenacity are weaker: | Yes | No |
